# Saline-push improves rubidium-82 PET image quality

**DOI:** 10.1007/s12350-018-1261-4

**Published:** 2018-03-27

**Authors:** Jennifer M. Renaud, Kai Yi Wu, Kimberly Gardner, May Aung, Rob S. B. Beanlands, Robert A. deKemp

**Affiliations:** National Cardiac PET Centre, University of Ottawa Heart Institute, 40 Ruskin Street, Ottawa, ON K1Y 4W7 Canada

**Keywords:** Rubidium-82, myocardial perfusion imaging, continuous quality improvement, positron emission tomography

## Abstract

**Introduction:**

Rubidium-82 (^82^Rb) PET is used widely for myocardial perfusion imaging. The purpose of this study was to investigate if an additional saline-push following the ^82^Rb elution improves PET image quality.

**Methods:**

^82^Rb PET scans were acquired with and without 26 mL saline-push in six patients as part of a clinical quality improvement program. Dynamic images were analyzed to measure the total activity delivered to the superior vena cava (SVC) and retained in the left ventricle (LV) myocardium. Tracer uptake images were used to measure blood background coefficient-of-variation (COV), myocardium-to-blood signal-to-noise ratio (SNR), and contrast-to-noise ratio (CNR) to assess image quality.

**Results:**

Similar eluted activity was measured with/without the saline-push (830 vs 795 MBq; *P* = 0.24). The activity delivered to the heart and retained in the myocardium was consistently increased more than twofold (SVC: + 114% and LV: + 104%; *P* < 0.001) with the saline-push. Image quality was improved in all patients, with lower background noise (COV: − 19%), higher SNR (+ 24%) and CNR (+ 27%) (all *P* ≤ 0.01).

**Conclusions:**

The saline-push used to flush ^82^Rb activity out of the infuser tubing, patient injection and intravenous access lines consistently increased the activity delivered to the heart by twofold. This technique is recommended to maximize image quality with ^82^Rb PET.

**Electronic supplementary material:**

The online version of this article (10.1007/s12350-018-1261-4) contains supplementary material, which is available to authorized users.

## Introduction

**Figure 1:**
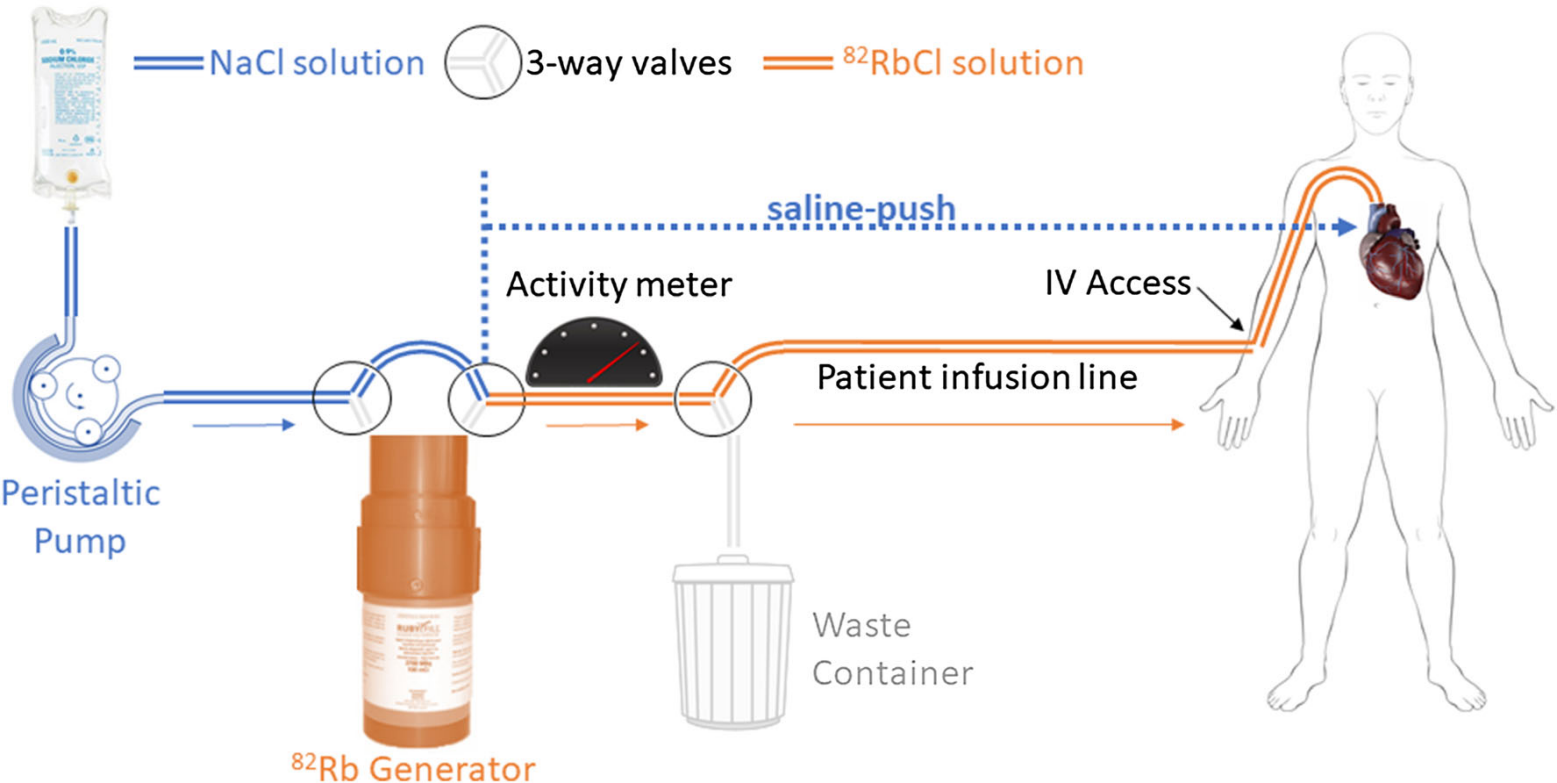
Three-way valves are used to ‘bypass’ the ^82^Rb generator, allowing saline flush (blue) of the infuser tubing from the generator to the patient infusion and intravenous (IV) access lines (orange), and to push the eluted ^82^Rb activity through the peripheral and central veins to the heart

Rubidium-82 (^82^Rb) is the most commonly used PET tracer for stress myocardial perfusion imaging (MPI) and is increasingly used for quantification of myocardial blood flow and stress/rest flow reserve.^1^ The ultra-short half-life of 76 seconds requires careful timing of the tracer infusion with the start of PET imaging for optimal and reproducible image quality. However, this can be challenging in practice as the parent Strontium-82 (^82^Sr) activity in the ^82^Sr/Rb generator decays over several weeks, causing the volume of saline eluate and the corresponding time for elution to increase for the same prescribed activity. This results in additional decay of the ^82^Rb activity during the tracer elution, which can reduce image quality particularly near the end of the generator shelf-life. Recently, a new ^82^Rb elution system has become available which allows consistent administration of ^82^Rb activity regardless of the generator age.^2^–^4^ This system normally includes an automated post-elution flush (saline-push) of the infuser tubing and patient injection line (6 mL) to help deliver all the eluted activity to the patient intravenous (IV) site, as well as an additional (+ 20 mL) saline volume to help decrease the transit-time from the IV access site to the superior vena cava and right atrium of the heart (Figure 1). The purpose of this study was to evaluate the impact of this added saline-push on ^82^Rb PET image quality.

## Methods

As part of the routine clinical quality improvement program at our institution, six scans (4 rest + 2 stress) were identified during a 4-month period (Sep to Dec 2017) with premature termination of the rubidium injection, before the start of the normal saline-push.^4^ In these scans (−push), the flow of saline was stopped (aborted) in error, immediately after the prescribed total activity was eluted from the ^82^Rb generator, and without administration of the standard saline-push volume. As such, the infuser tubing from the generator to the patient infusion line and IV access site were not flushed normally, and tracer transit to the heart was reliant only on the venous return blood flow. Due to clinical concern about the potential impact on image quality with these aborted injections, a repeat injection and scan (+push) was performed immediately following the first scan; the same activity was prescribed and then delivered normally including the standard saline-push volume (26 mL) to flush the infuser tubing, patient infusion, and IV lines as well as the cephalic and saphenous veins. The imaging protocol is illustrated in Figure S1 (online supplement). Patients provided written informed consent as part of the REST-PET trial (NCT02117284).

List-mode acquisitions were started at the time of tracer injection (30 s ‘square-wave’ at constant-activity-rate of 0.3 MBq/kg/s = 9 MBq/kg) at rest or following dipyridamole stress (140 μg/min/kg), according to current guidelines and local practice.^5^,^6^ Dynamic PET images (9 × 10 s, 3 × 30 s, 1 × 60 s = 4 min total) were acquired in rapid succession (∆*t* = 6 ± 1 min) first without and then with the saline-push on a Discovery 690 PET-VCT scanner (GEHC, Waukesha, WI). Dynamic images were reconstructed using the vendor iterative method (VuePoint HD) with 8-mm post-reconstruction Hann filter. Total counts (prompt − random coincidences) were recorded during the tracer uptake phase (2-4 min) and used to reconstruct static (ungated) images, then smoothed with 12-mm Hann post-filter. The aborted elution (− push) scans were shortened compared to our normal practice (4 vs 8 min) to minimize inter-scan delay, particularly during stress to ensure that the repeated clinical scan was acquired near peak hyperemia. The repeated elution (+ push) scans were analyzed using the same 4-min data to avoid any bias in image quality that would arise from a longer scan.

As illustrated in Figure S2A (online supplement), arterial blood time-activity curves (TAC) were measured using a volume-of-interest (VOI) placed in the superior vena cava (SVC), to compare the delivered ^82^Rb activity profiles with and without the saline-push. The SVC curve was also integrated over time, to assess the total activity (MBq·min) delivered to the heart over the course of the 4-minute scan. TAC values in the left ventricle (LV) myocardium were measured using FlowQuant® automated processing as previously described.^6^ Background noise was measured as the coefficient-of-variation (COV = SD/mean) of activity values in a spherical VOI placed in the left atrium (LA) blood pool as shown in Figure S2B (online supplement). The myocardium (LV) and blood background (COV) values measured during the uptake phase were used to calculate ^82^Rb image signal-to-noise ratios (SNR) and contrast-to-noise ratios (CNR) for each scan according to Equations 1 and 2 below.

1 $$ {\text{SNR}} = {\text{LV}} \div {\text{COV}} $$

2 $$ {\text{CNR}} = ({\text{LV}} - {\text{LA}}) \div {\text{COV}}. $$

## Results

Patient demographics and ^82^Rb activity elution data are summarized in Table 1. The mean patient age (65 years) and weight (94 kg) are typical of those referred for ^82^Rb PET MPI at our institution. The total injected saline volume was 29 ± 3 mL higher for elutions with vs without the saline-push, although the activity eluted from the generator was not significantly different between the repeat injections (830 vs 795 MBq; *P* = 0.24). The differences in volume injected with vs without the saline-push were slightly higher than the 26 mL expected in most patients, likely due to incomplete recharging of the generator activity in the 5- to 6-min time interval between elutions.

**Table 1:** Patient and rubidium elution data

Num	Sex	Scan type	Patient age (years)	Patient weight (kg)	LVEF (%)	Activity delivered (− push)	Activity delivered (+ push)	Volume delivered (− push)	Volume delivered (+ push)	Time interval (∆min)
1	M	Rest	59	138	25	980	1150	20	55	6
2	F	Rest	75	104	66	937	942	11	39	6
3	M	Stress	75	70	29	633	648	11	39	5
4	M	Rest	65	73	48	652	663	11	39	6
5	M	Stress	54	80	60	709	718	12	39	5
6	M	Rest	64	95	65	856	860	10	36	9
Mean ± Std Dev			65 ± 8	94 ± 25	49 ± 18	795 ± 150	830 ± 195	13 ± 4	41 ± 7	6 ± 1

**Figure 2:**
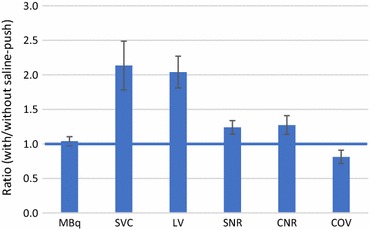
Effects on ^82^Rb image quality with vs without saline-push. Total ^82^Rb activity (MBq) eluted from the generator was unchanged, but the activity delivered to the heart (SVC) and retained in the LV myocardium increased by more than 2-fold. LV-to-blood SNR and CNR increased by 24% and 27%, with concordant decrease in the blood background COV by 19% (all *P* ≤ 0.01). Error bars are ± 95% confidence intervals of the mean

The total coincidence counts recorded during the uptake phase of the PET scans were twofold higher using the saline-push (31.4 ± 5.5 vs 15.6 ± 2.1 million counts; *P* < 0.001). Despite no measured difference in the eluted activity (MBq) from the generator, the integral activity delivered to the heart (SVC) increased more than 2-fold using the saline-push (*P* < 0.001), as shown in Figure 2. The average ^82^Rb activity retained by the heart (LV myocardium) was also increased by 2-fold (*P* < 0.001), as shown individually for all 6 patients in Figure S3 (online supplement).

**Figure 3:**
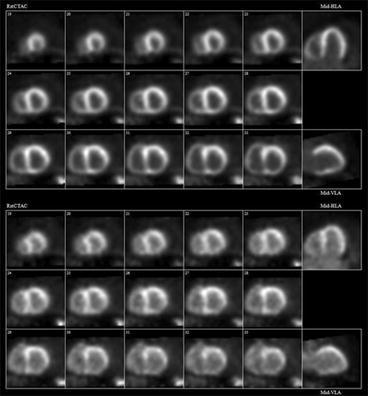
^82^Rb PET perfusion images at rest in patient #1 (59-year-old male, 138 kg, BMI = 48, LVEF = 25%) obtained with (**A**) and without (**B**) the additional saline-push following elution of the prescribed activity from the generator. Image quality and myocardium-to-blood contrast are improved markedly using the saline-push

The average image SNR and CNR were both improved in the LV myocardium by approximately 25% using the saline-push (*P* = 0.002 and 0.01, respectively) as shown in Figure 2, and for all patients individually in Figure S4 (online supplement). The improvement in image quality was also associated with a similar change in the LA blood background noise (COV) which decreased by approximately 20% (*P* = 0.01). The effects of the saline-push on ^82^Rb image quality are shown qualitatively for one patient in Figure 3. This patient had the lowest left ventricle ejection fraction (LVEF) as shown in Table 1. Analysis of the changes in CNR, SNR, and background noise COV with LVEF (Figure S5) demonstrated a significant effect of LVEF on improved SNR (*P* = 0.034) and a trend for reduced COV (*P* = 0.078), suggesting that the benefits of the saline-push may be greater in patients with lower LVEF.

## Discussion

As one of the two FDA-approved myocardial perfusion tracers, ^82^Rb PET is a robust tool for diagnosis, risk stratification, and management of patients with suspected or known coronary artery disease (CAD).^7^ The useful shelf-life of the ^82^Rb generator is typically determined by the minimum activity available for patient injection, which decreases as the parent ^82^Sr isotope decays over time (25.5-day half-life). PET image quality (SNR) is directly related to the number of recorded counts during the uptake phase.^8^ In this small study, we have demonstrated the consistent and highly significant increase in recorded counts and image quality obtained using ~ 25 mL saline-push without increasing the ^82^Rb activity eluted from the generator. This volume of saline is similar to that used routinely to optimize image quality during contrast administration for x-ray CT coronary angiography.^9^ The ^82^Rb activity delivered to the heart and retained in the myocardium was consistently increased more than 2-fold, which provides tremendous opportunity to increase image quality or to extend the useful shelf-life of the ^82^Rb generator. This additional activity is a result of 2 separate processes: i. flushing the eluate in the tubing volume between the generator and the patient IV access site, and ii. decreasing the ^82^Rb isotope decay-time during venous transit from the IV access site to the heart.

The measured increase in SNR of ~ 25% is consistent with the ~ 20% decrease in image noise (i.e., 1.25 = 1/0.8) but is somewhat less than the theoretical improvement (√2) expected from a 2-fold increase in counts.^8^ This is likely due to the use of statistical iterative reconstruction and image smoothing, which also have independent effects to improve image quality, compared to filtered-backprojection without the use of an apodizing window of the ramp-filter. The improvements in image quality were measured using a relatively short (4 min) scan time compared to the typical 8-min dynamic scan duration used clinically at our institution. The 4-min period used for analysis was the longest common time available to compare the properties of the aborted and repeated scans. While the exact numerical results may differ slightly, the benefits of the saline-push should translate directly to the longer scan times, since 70% of the counts are acquired in the first 2 min of the uptake phase, i.e., 2-4 min after the scan starts.

In the present study, an integrated ‘bypass line’ was used to administer the saline-push automatically following elution of the ^82^Rb generator.^4^ However, similar results may be expected with other generator systems using a manual flush, which could be administered by inserting a 3-way valve and flush-syringe at the junction between the generator tubing and the patient infusion line. If the saline-push is administered manually, adequate shielding should be employed to minimize radiation exposure to the imaging technologist. The patient benefits from improvement in image quality should be balanced against the risk of increased occupational exposure.

While the current data were acquired using the ‘constant-activity-rate’ mode of infusion,^4^ the effects of the saline-push should apply generally to other infusion modes, e.g., constant-flow-rate used with this or other rubidium generator systems and may help overcome the practical difficulties of weight-based dosing reported for ^82^Rb PET.^10^ The saline flow-rate is not changed during any of the patient infusions, even in the constant-activity-rate mode, therefore the time-delay to flush activity from the generator tubing and patient infusion lines will be similar for a given flow-rate regardless of the mode of tracer infusion. For other PET perfusion tracers such as ^13^N-ammonia or ^15^O-water, a dual-syringe pump could be used to automatically deliver the saline-push following tracer administration, again similar to methods used routinely for coronary CT angiography.^9^

## Conclusion

An additional 25-30 mL saline-push is recommended following elution of the prescribed activity from a ^82^Rb generator to flush the infuser tubing, patient infusion, and intravenous access lines, and to increase venous return to the heart. This simple technique increases myocardial uptake by a factor of 2, and image SNR and CNR by 25%.

## New knowledge gained

^82^Rb PET myocardial perfusion image quality is significantly improved by flushing all of the eluted activity out of the infuser tubing and patient IV lines, followed by an additional 20 mL saline-push to accelerate the tracer transit to the heart. The benefits to improve image quality may be highest in patients with low cardiac output, where delayed clearance from the blood pool is often observed.

## Electronic supplementary material

Below is the link to the electronic supplementary material.

Supplementary material 1 (PPTX 429 kb)

Supplementary material 2 (DOCX 2529 kb)

## Abbreviations

CNR: Contrast-to-noise ratio
COV: Coefficient-of-variation
LA: Left atrium
LV: Left ventricle
LVEF: Left ventricle ejection fraction
MBq: Megabecquerel
MPI: Myocardial perfusion imaging
PET: Positron emission tomography
^82^Rb: Rubidium-82
SNR: Signal-to-noise ratio
SVC: Superior vena cava
