# Supplementary material for: Saline-push improves rubidium-82 PET image quality
Source: J Nucl Cardiol. 2018 Mar 27;26(6):1869–74. doi: 10.1007/s12350-018-1261-4 (PMC6908549; doi:10.1007/s12350-018-1261-4)
Supplement: Supplementary file 1 — Supplementary material 1 (PPTX 429 kb) [file 12350_2018_1261_MOESM1_ESM.pptx]

## Slide 1
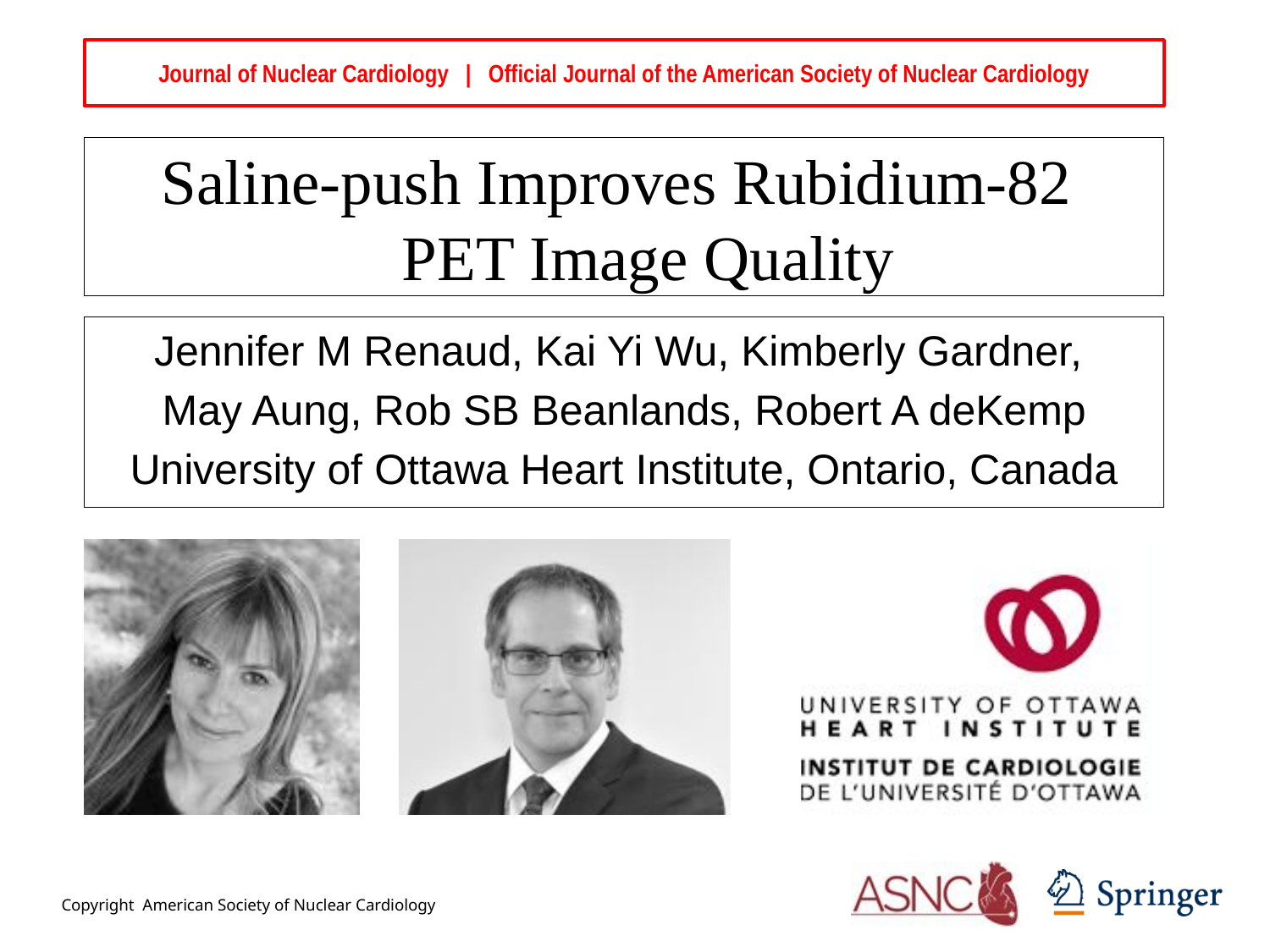

Journal of Nuclear Cardiology | Official Journal of the American Society of Nuclear Cardiology
# Saline-push Improves Rubidium-82 PET Image Quality
Jennifer M Renaud, Kai Yi Wu, Kimberly Gardner,
May Aung, Rob SB Beanlands, Robert A deKemp
University of Ottawa Heart Institute, Ontario, Canada
Institution
Picture/Logo
Optional
Copyright American Society of Nuclear Cardiology

## Slide 2
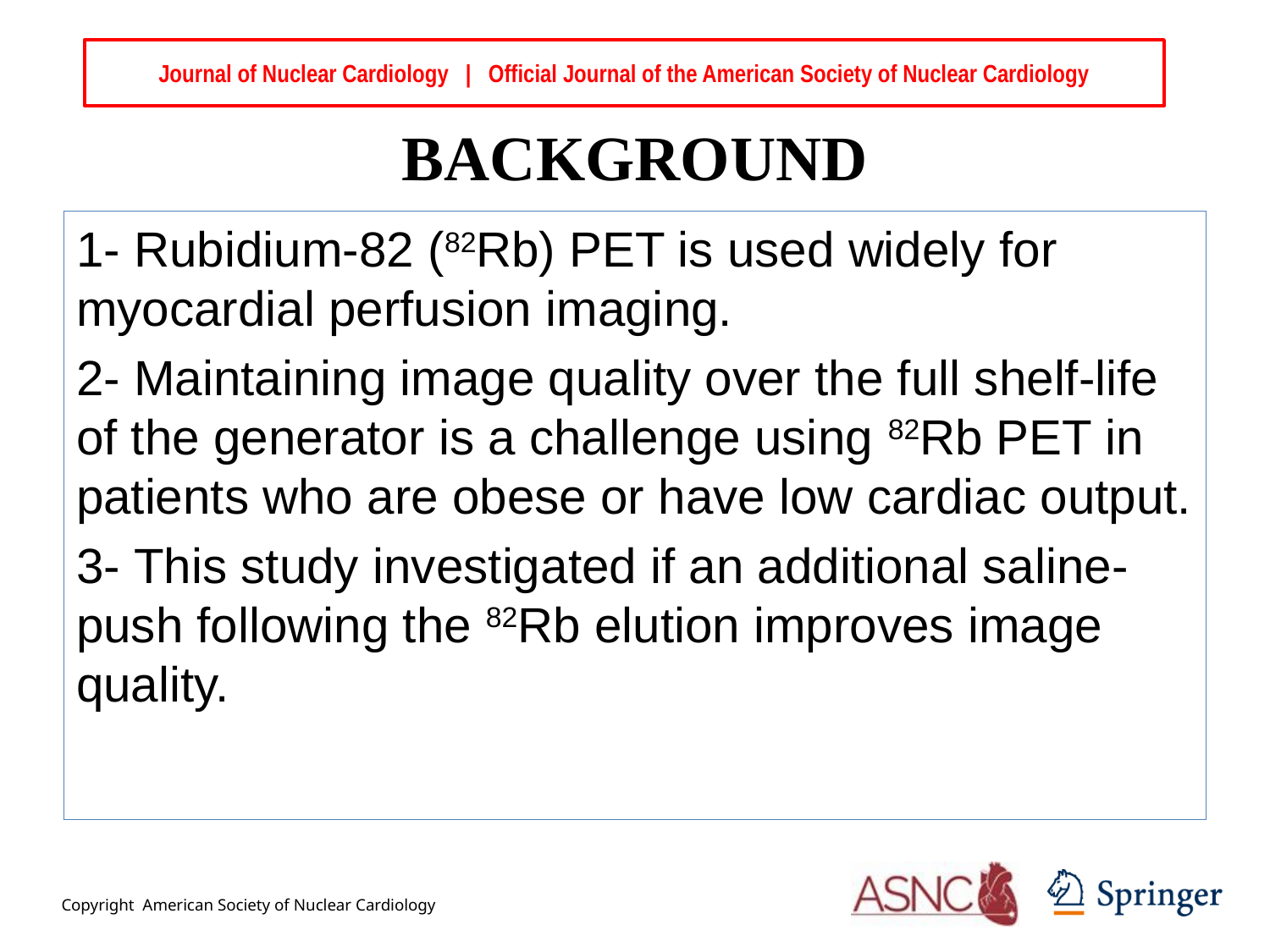

Journal of Nuclear Cardiology | Official Journal of the American Society of Nuclear Cardiology
# BACKGROUND
1- Rubidium-82 (82Rb) PET is used widely for myocardial perfusion imaging.
2- Maintaining image quality over the full shelf-life of the generator is a challenge using 82Rb PET in patients who are obese or have low cardiac output.
3- This study investigated if an additional saline-push following the 82Rb elution improves image quality.
Copyright American Society of Nuclear Cardiology

## Slide 3
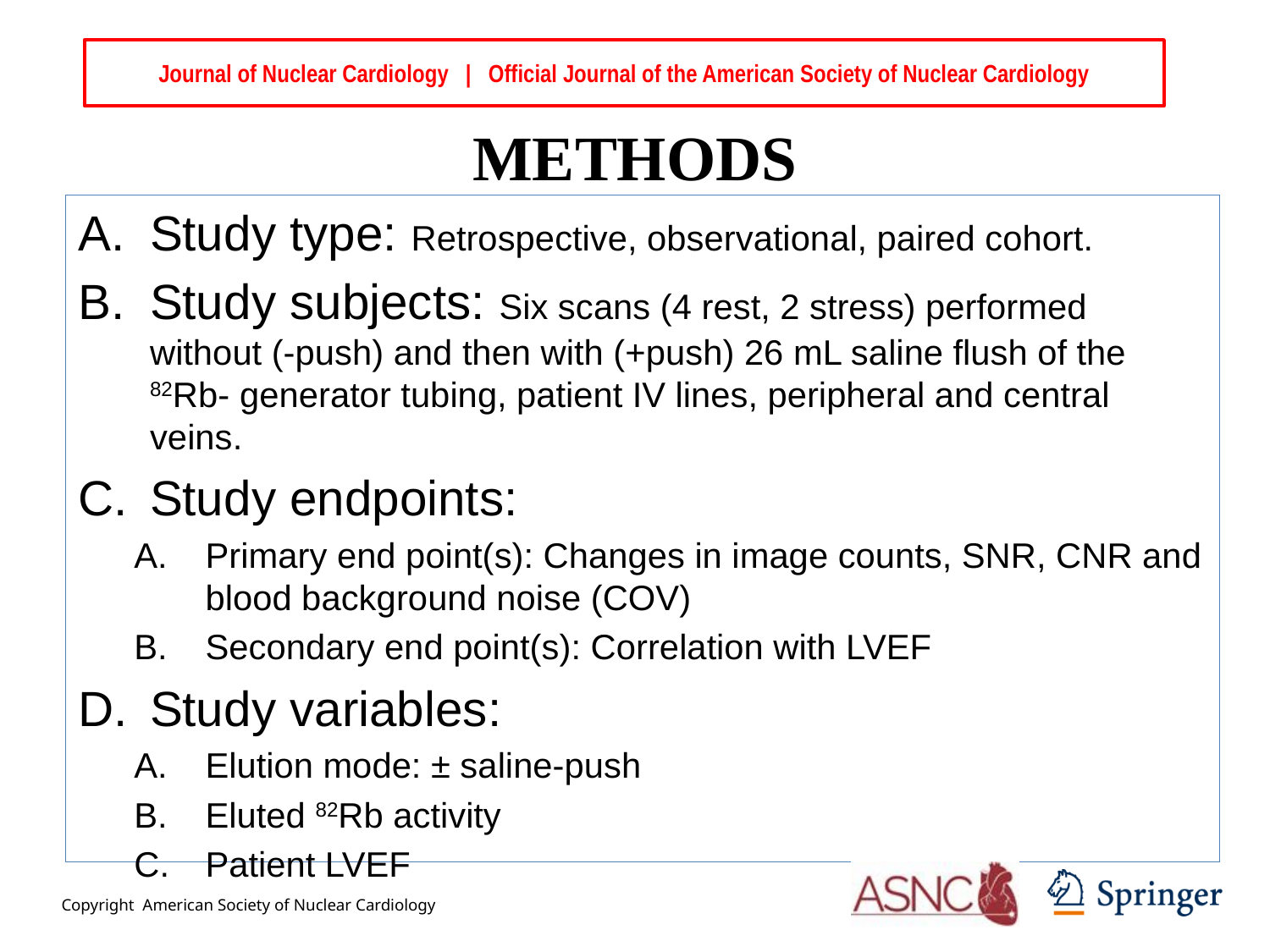

Journal of Nuclear Cardiology | Official Journal of the American Society of Nuclear Cardiology
# METHODS
Study type: Retrospective, observational, paired cohort.
Study subjects: Six scans (4 rest, 2 stress) performed without (-push) and then with (+push) 26 mL saline flush of the 82Rb- generator tubing, patient IV lines, peripheral and central veins.
Study endpoints:
Primary end point(s): Changes in image counts, SNR, CNR and blood background noise (COV)
Secondary end point(s): Correlation with LVEF
Study variables:
Elution mode: ± saline-push
Eluted 82Rb activity
Patient LVEF
Copyright American Society of Nuclear Cardiology

## Slide 4
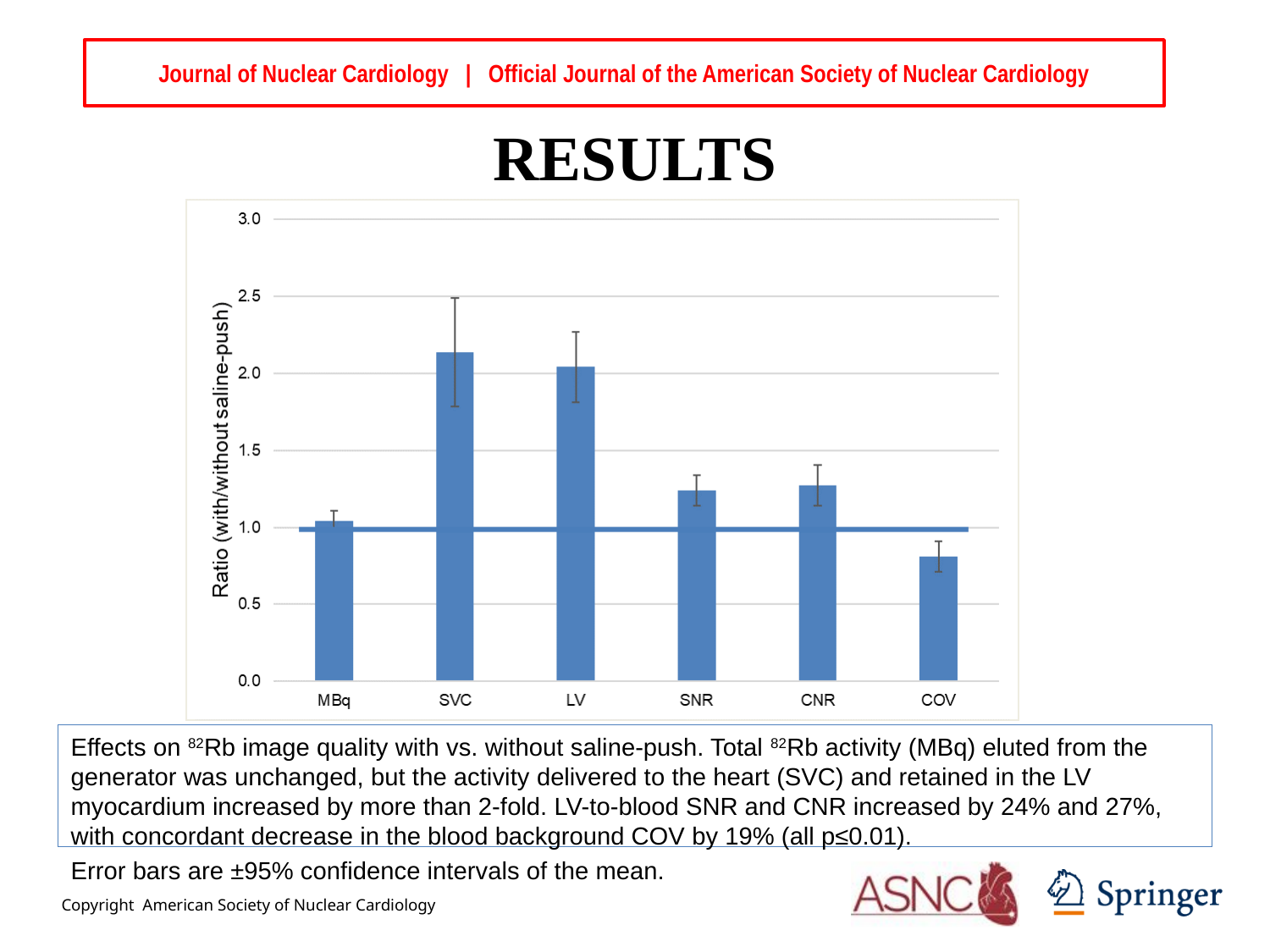

Journal of Nuclear Cardiology | Official Journal of the American Society of Nuclear Cardiology
# RESULTS
Effects on 82Rb image quality with vs. without saline-push. Total 82Rb activity (MBq) eluted from the generator was unchanged, but the activity delivered to the heart (SVC) and retained in the LV myocardium increased by more than 2-fold. LV-to-blood SNR and CNR increased by 24% and 27%, with concordant decrease in the blood background COV by 19% (all p≤0.01).
Error bars are ±95% confidence intervals of the mean.
Copyright American Society of Nuclear Cardiology

## Slide 5
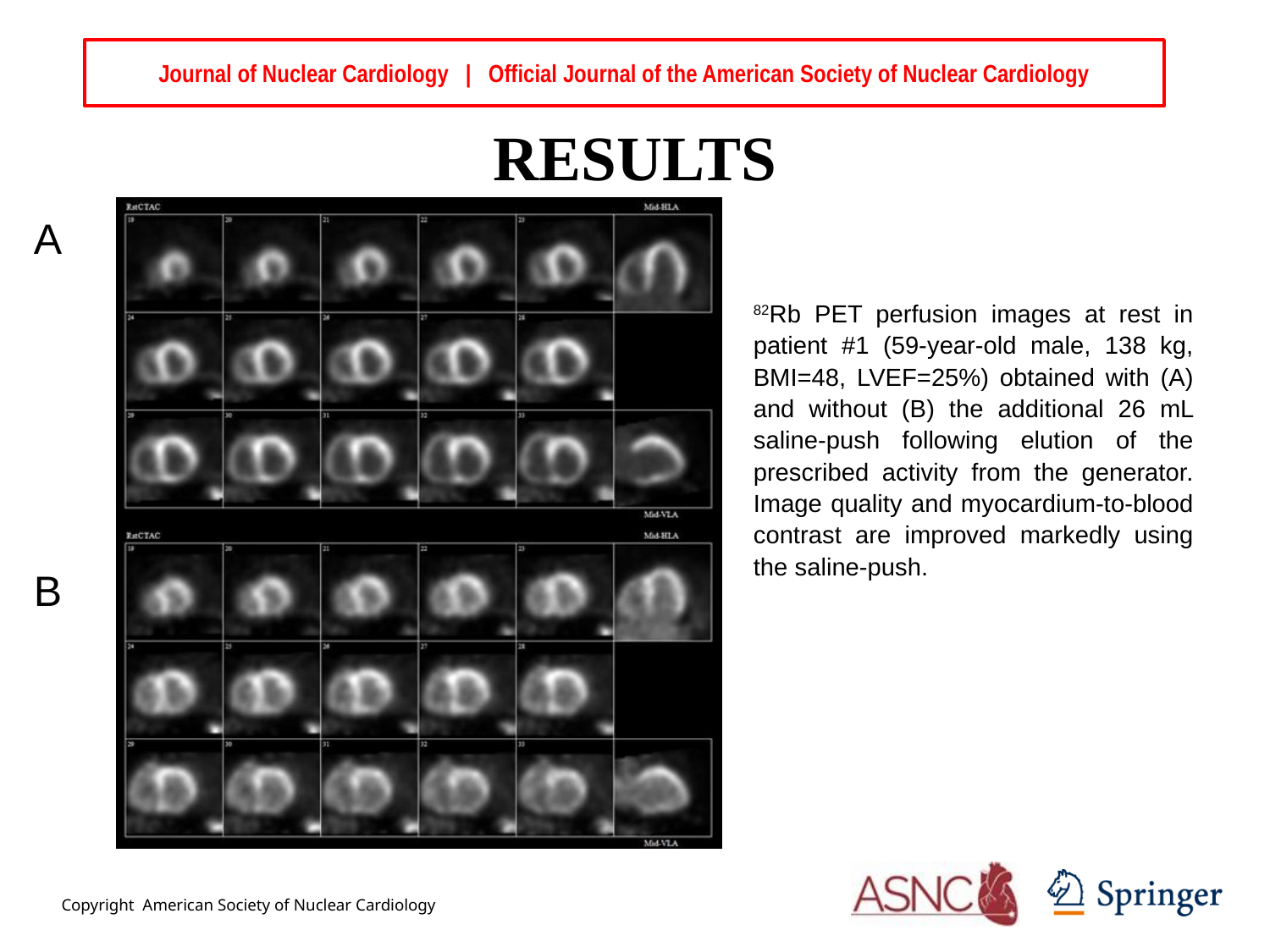

Journal of Nuclear Cardiology | Official Journal of the American Society of Nuclear Cardiology
# RESULTS
A
82Rb PET perfusion images at rest in patient #1 (59-year-old male, 138 kg, BMI=48, LVEF=25%) obtained with (A) and without (B) the additional 26 mL saline-push following elution of the prescribed activity from the generator. Image quality and myocardium-to-blood contrast are improved markedly using the saline-push.
B
Copyright American Society of Nuclear Cardiology

## Slide 6
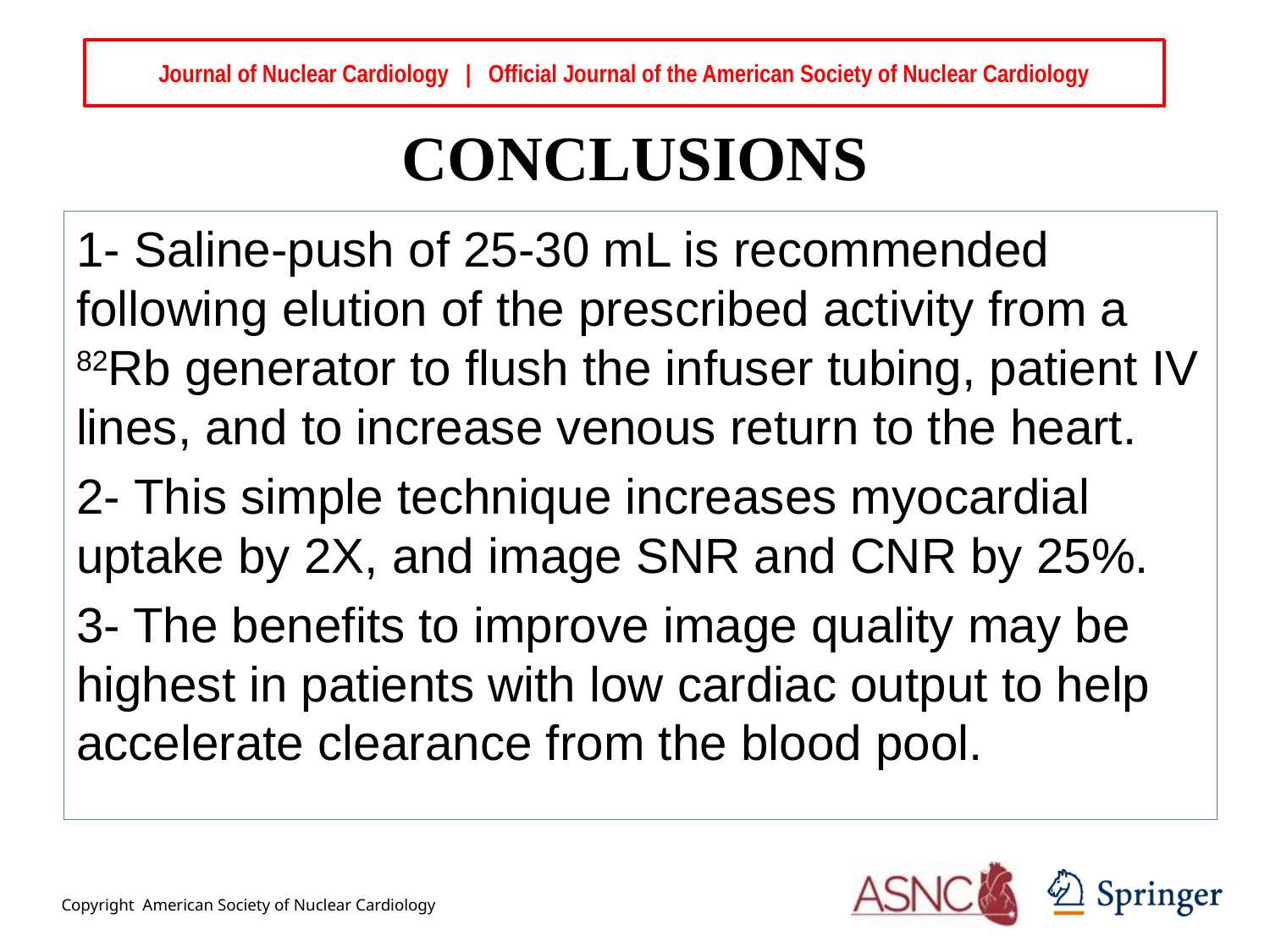

Journal of Nuclear Cardiology | Official Journal of the American Society of Nuclear Cardiology
# CONCLUSIONS
1- Saline-push of 25-30 mL is recommended following elution of the prescribed activity from a 82Rb generator to flush the infuser tubing, patient IV lines, and to increase venous return to the heart.
2- This simple technique increases myocardial uptake by 2X, and image SNR and CNR by 25%.
3- The benefits to improve image quality may be highest in patients with low cardiac output to help accelerate clearance from the blood pool.
Copyright American Society of Nuclear Cardiology
