# Supplementary material for: Saline-push improves rubidium-82 PET image quality
Source: J Nucl Cardiol. 2018 Mar 27;26(6):1869–74. doi: 10.1007/s12350-018-1261-4 (PMC6908549; doi:10.1007/s12350-018-1261-4)
Supplement: Supplementary file 2 — Supplementary material 2 (DOCX 2529 kb) [file 12350_2018_1261_MOESM2_ESM.docx]

**Online Supplemental Materials:**

**
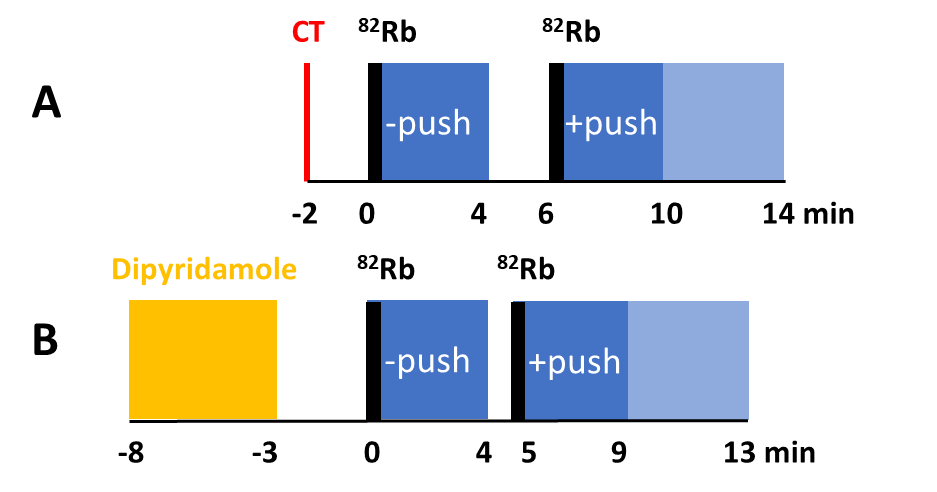
**

**Figure S1.** PET imaging protocol at rest (A) and stress (B). 4-minute dynamic scans were acquired after the aborted elutions (−push) followed immediately by a repeated elution and scan (+push) 1 or 2 min later at stress or rest, respectively. The repeated scans (+push) were 8 min long according to routine clinical practice, but only the first 4 min were analyzed to allow unbiased comparison to the −push scan data.

**A.**


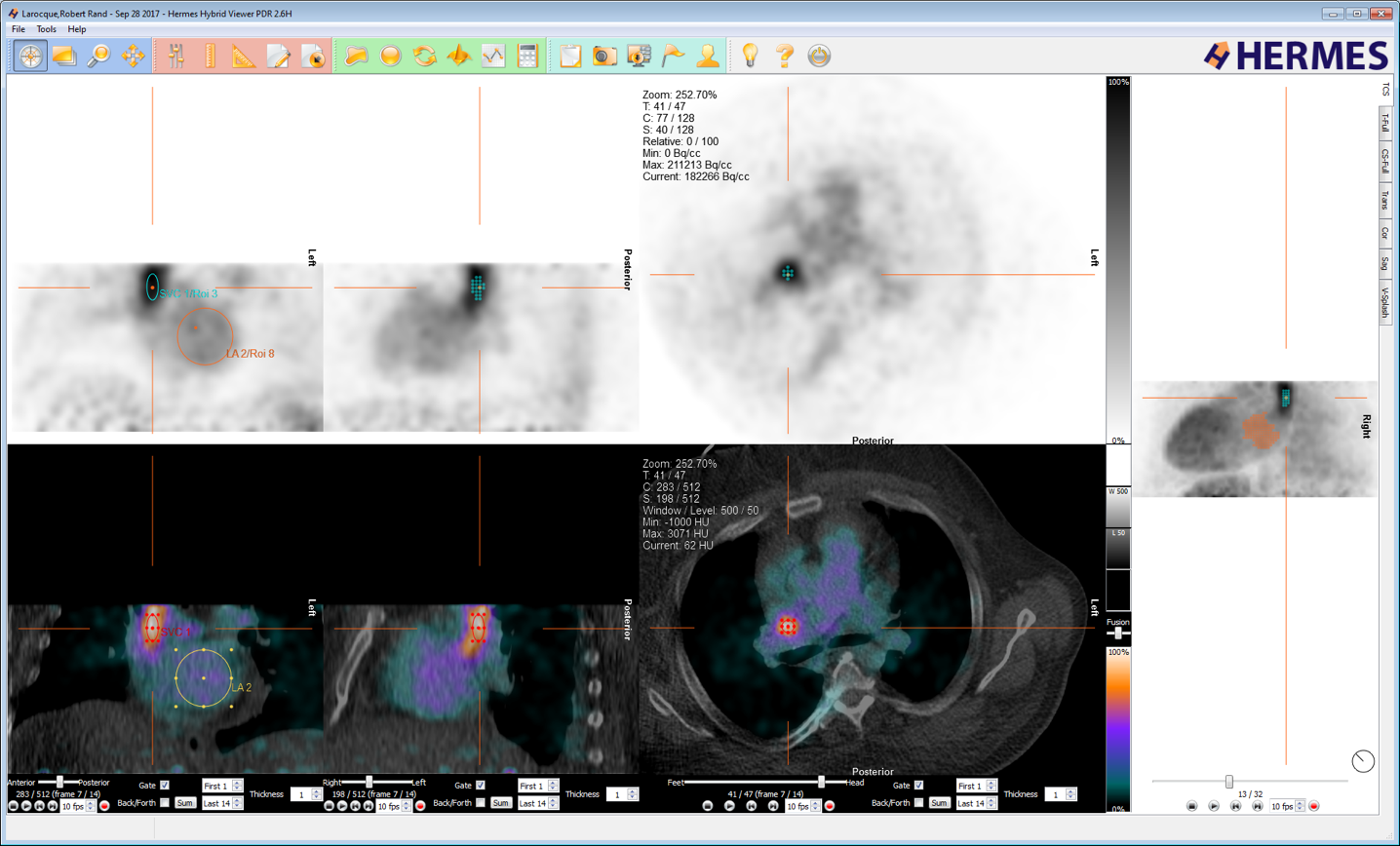


**B.**


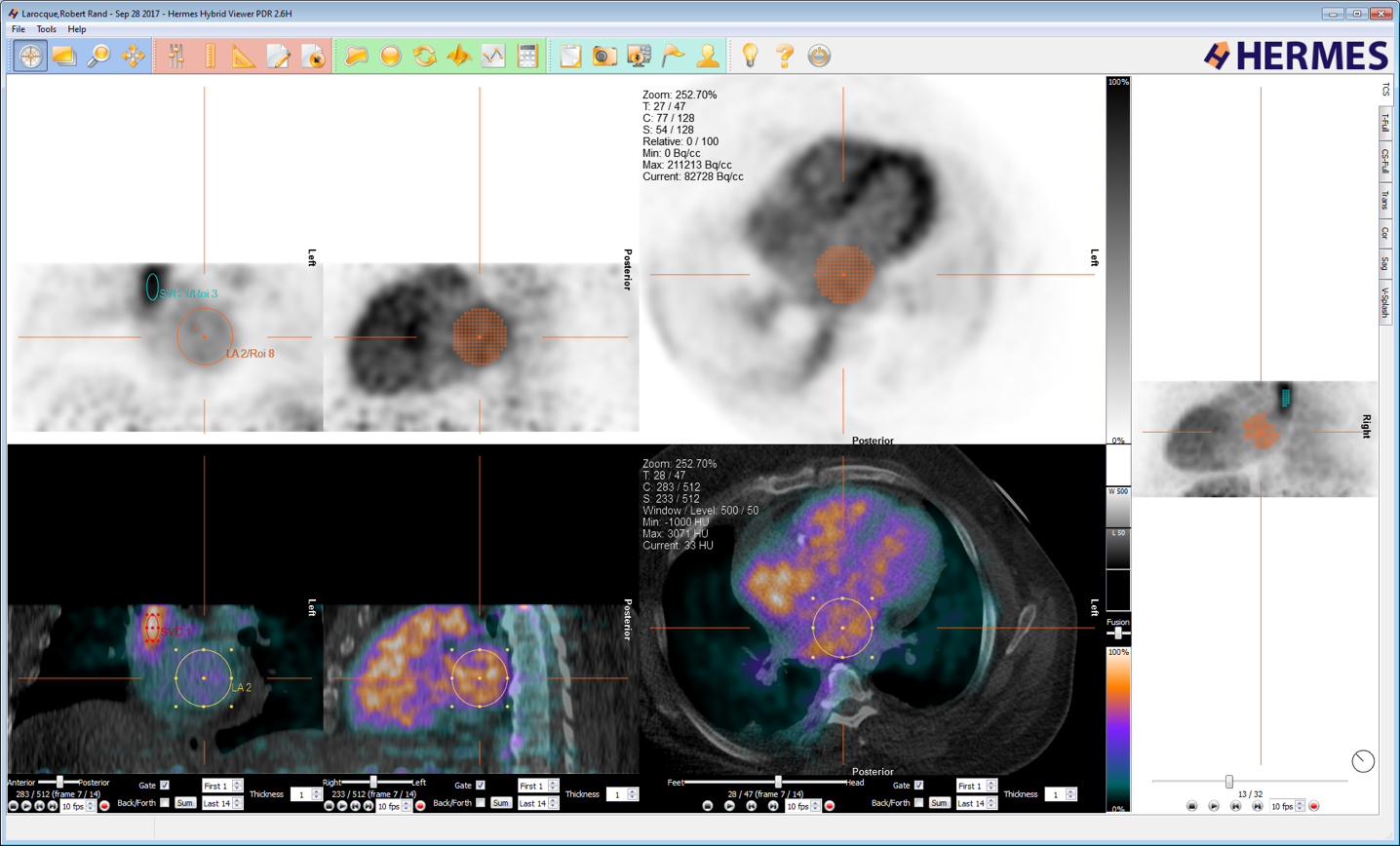


**Figure S2.** ^82^Rb PET cross-sectional and maximum intensity projection (greyscale) and PET-CT fusion (color) images are shown for patient #1: **A.** in the superior vena cava (SVC) and **B.** in the left atrium (LA). Volumes-of-interest (VOI) were drawn in the SVC as shown in red on the fused PET-CT images, and cyan on the PET images. Volumes-of-interest (VOI) were drawn in the LA cavity as shown in yellow on the fused PET-CT images, and orange on the PET images. Time-activity-curves were measured in these regions using HybridViewer software v1.4 (HERMES Medical Solutions, Sweden)

| A.  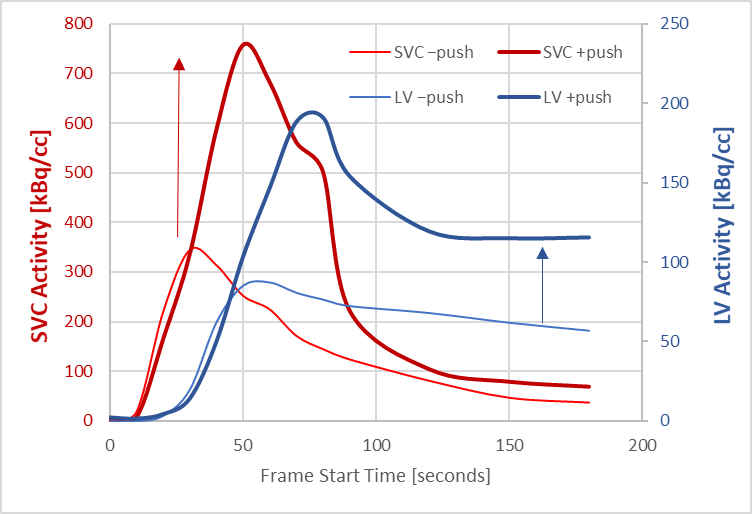 | B.  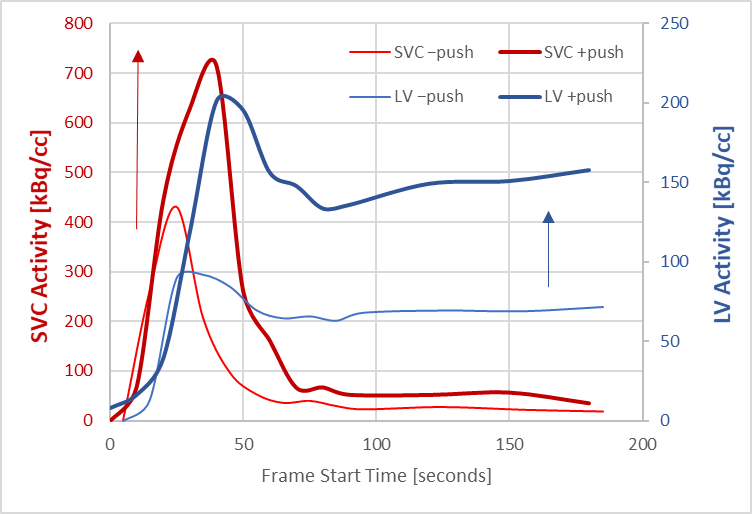 |
| --- | --- |
| C.  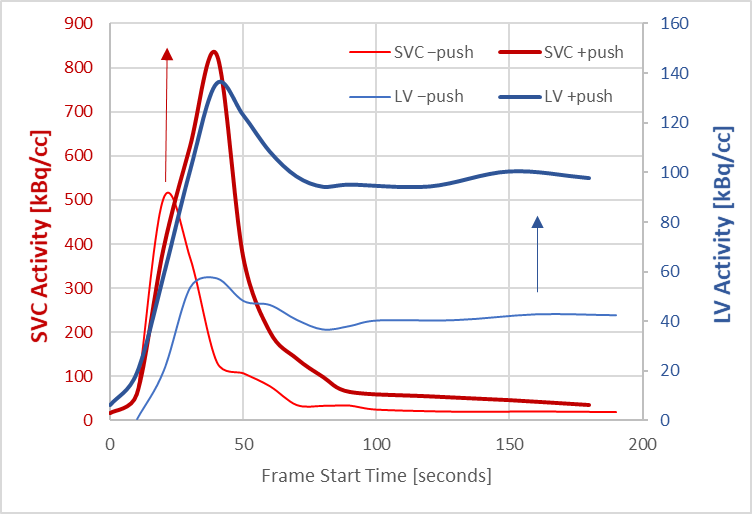 | D.  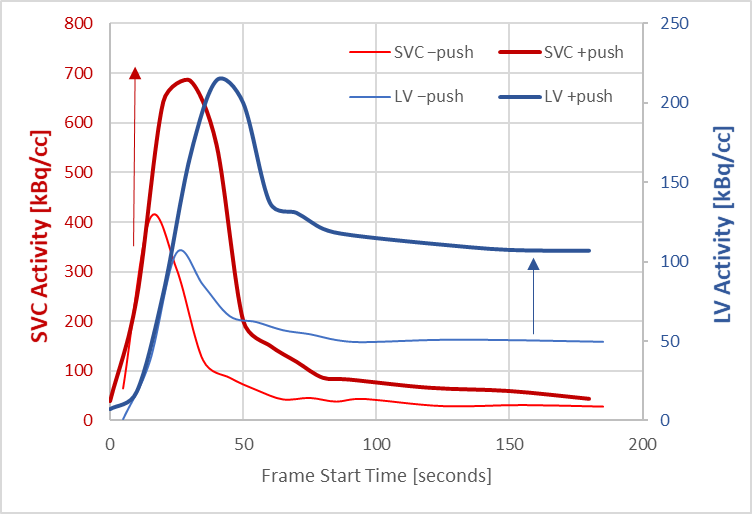 |
| E.  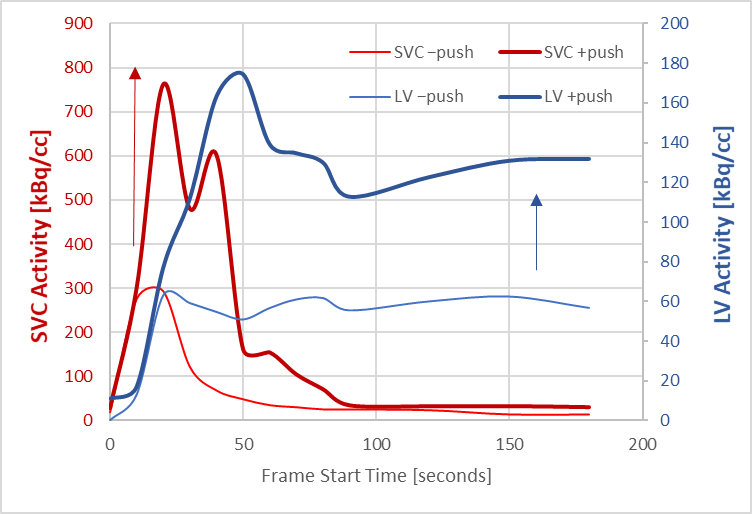 | F.  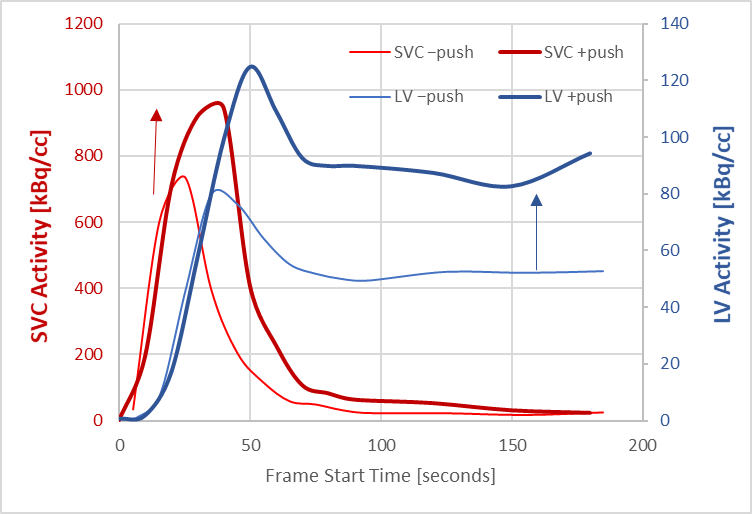 |

**Figure S3.** Time-activity curves in the SVC and LV myocardium for ^82^Rb PET scans in patients 1 to 6 (A to F), with (+push) and without (−push) saline-push following the elution of activity from the generator.

| A. | B. | C. |
| --- | --- | --- |
| D. | E. | F. |


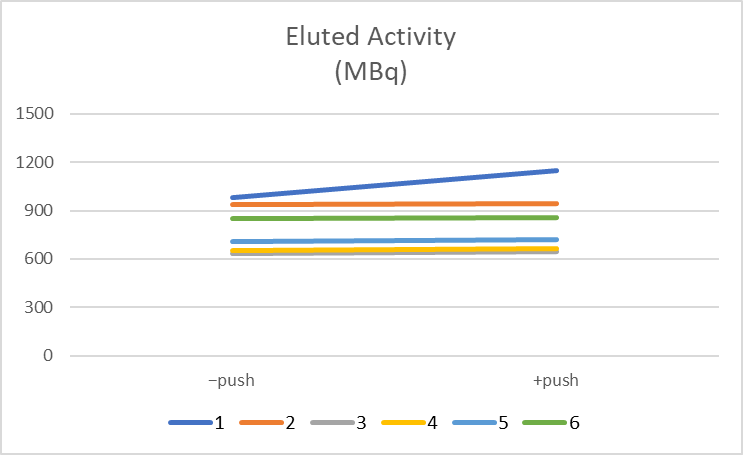
**Figure S4.** Effects on ^82^Rb activity and image quality with (+push) and without (−push) saline-push in 6 patients:


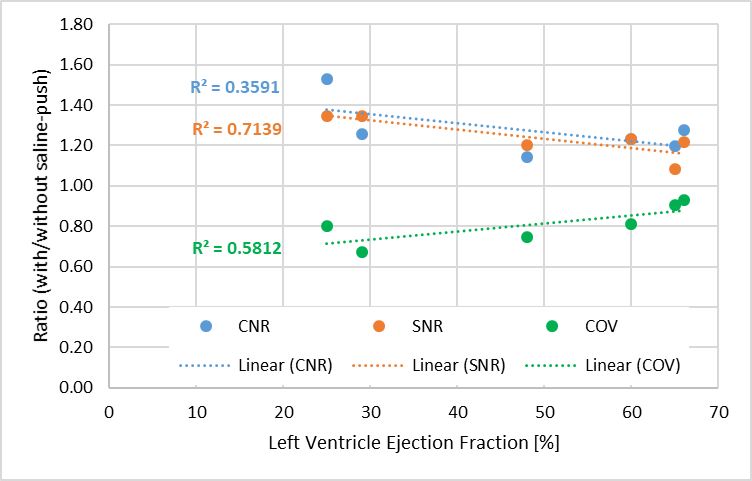


**Figure S5.** Changes in myocardial contrast-to-noise (CNR), myocardial signal-to-noise (SNR) and left atrial blood background coefficient-of-variation (COV) as a function of left ventricle ejection fraction (LVEF). The correlation of SNR with LVEF was statistically significant (p=0.034).
